# Prevalence of prenatal zinc deficiency and its association with socio-demographic, dietary and health care related factors in Rural Sidama, Southern Ethiopia: A cross-sectional study

**DOI:** 10.1186/1471-2458-11-898

**Published:** 2011-11-29

**Authors:** Samson Gebremedhin, Fikre Enquselassie, Melaku Umeta

**Affiliations:** 1 College of Agriculture, Hawassa University, Hawassa, Ethiopia; 2 School of Public Health, Addis Ababa University, Addis Ababa, Ethiopia; 3 School of Medicine, Addis Ababa University, Addis Ababa, Ethiopia

**Keywords:** Maternal nutrition, Zinc deficiency, Serum zinc concentration

## Abstract

**Background:**

Several studies witnessed that prenatal zinc deficiency (ZD) predisposes to diverse pregnancy complications. However, scientific evidences on the determinants of prenatal ZD are scanty and inconclusive. The purpose of the present study was to assess the prevalence and determinants of prenatal ZD in Sidama zone, Southern Ethiopia.

**Methods:**

A community based, cross-sectional study was conducted in Sidama zone in January and February 2011. Randomly selected 700 pregnant women were included in the study. Data on potential determinants of ZD were gathered using a structured questionnaire. Serum zinc concentration was measured using Atomic Absorption Spectrometry. Statistical analysis was done using logistic regression and linear regression.

**Results:**

The mean serum zinc concentration was 52.4 (+/-9.9) μg/dl (95% CI: 51.6-53.1 μg/dl). About 53.0% (95% CI: 49.3-56.7%) of the subjects were zinc deficient. The majority of the explained variability of serum zinc was due to dietary factors like household food insecurity level, dietary diversity and consumption of animal source foods. The risk of ZD was 1.65 (95% CI: 1.02-2.67) times higher among women from maize staple diet category compared to *Enset *staple diet category. Compared to pregnant women aged 15-24 years, those aged 25-34 and 35-49 years had 1.57 (95% CI: 1.04-2.34) and 2.18 (95% CI: 1.25-3.63) times higher risk of ZD, respectively. Women devoid of self income had 1.74 (95% CI: 1.11-2.74) time increased risk than their counterparts. Maternal education was positively associated to zinc status. Grand multiparas were 1.74 (95% CI: 1.09-3.23) times more likely to be zinc deficient than nulliparas. Frequency of coffee intake was negatively association to serum zinc level. Positive association was noted between serum zinc and hemoglobin concentrations. Altitude, history of iron supplementation, maternal workload, physical access to health service, antenatal care and nutrition education were not associated to zinc status.

**Conclusion:**

ZD is of public health concern in the area. The problem must be combated through a combination of short, medium and long-term strategies. This includes the use of household based phytate reduction food processing techniques, agricultural based approaches and livelihood promotion strategies.

## Background

Zinc is one of the essential trace elements and vital micronutrients with diverse physiologic and metabolic functions [1]. It participates in all major biochemical pathways and plays multiple roles in the cellular proliferation and differentiation [2]. It affects physical growth, immunity, reproductive function and neuro-behavioral development [2,3].

The effects of zinc on maternal health and pregnancy outcomes have been studied in multiple observational and interventional studies [3]. Studies linked the deficiency with a wide range of complications including pregnancy induced hypertension, premature rupture of membrane, placental abruption, prolonged labour, hemorrhage, infections, intrauterine growth retardation, low birth weight, congenital anomalies, increased neonatal morbidity and poor neurobehavioral development [3,4].

Research conducted over years suggested that zinc deficiency (ZD) is widespread public health problem in the world [5]. World Health Organization estimated that ZD affects 31%, 4-73% in various regions, of the world's population [5]. Currently there is scant reliable information regarding the magnitude of ZD in pregnant women [4]. However, studies conducted in Ethiopia [6], Malawi [7], Nigeria [8], India [9], Iran [10] and Indonesia [11] reported high prevalence figures.

In 2004 International Zinc Nutrition Consultative Group (IZiNCG) estimated that 21.1% of Ethiopian population was at risk of inadequate dietary zinc intake [2]. However empirical evidences are scanty. A study conducted in Sidama zone reported 72% prevalence of ZD among pregnant women [6]. Whereas, a study in Addis Ababa found 0% deficiency and 11.3% marginal deficiency among lactating mothers [12].

This study was conducted in rural Sidama zone where seriously high prevalence of ZD was previously reported by relatively small scale surveys [6,13]. The study aims at assessing the prevalence and determinants of maternal ZD.

## Methods

### Study design

This is community based, cross-sectional, quantitative study with descriptive and analytic designs.

### Setting

The study was conducted in January and February 2011 in 18 kebeles of Sidama zone, Southern Ethiopia. The period was selected as it was neither food insecured nor harvest season. A kebele is the smallest administrative unit in Ethiopia comprising approximately 1000 households. Sidama zone is one of the 15 zones of Southern Nations Nationalities Peoples Region (SNNPR) [14]. The zone has population of 2,966,652 and population density of 430 people/km^2 ^[14]. It is characterized by three agro-ecological zones. The lowlands (20%), the midlands (50%) and the highlands (30%) [15]. About 85% of the population livelihood depends on subsistent farming [16]. Major crops grown in the area are *enset *(*Enset ventricosum*), coffee and maize [15]. The average rural household has 0.3 ha of land [16]. In the SNNPR access to health care is limited [17].

### Sample size

Sample size adequate for estimating the prevalence of ZD was computed using single proportion sample size calculation formula with the inputs of 95% confidence level, 5% of margin of error, design effect of 2, non-response rate of 10% and expected prevalence of ZD of 72% [6]. Accordingly, sample size of 682 was computed. However, in order to maximize the sample size for the analytic study component, 750 pregnant women were included in the study.

The adequacy of the sample size for investigating the key determinants of ZD (parity, maternal age, and gestational age) was assessed via double proportion sample size calculation formula using an online application [18]. The calculation was made based on the inputs of 95% confidence level, 80% study power and 1:1 ratio between cases and controls. Expected prevalence figures of the exposure factors in cases and controls were taken from studies conducted elsewhere [10,19]. Ultimately, the available sample size was judged to be adequate to study the aforementioned determinants.

### Sampling technique

Initially all the kebeles in the zone were listed and stratified into the three agro-ecological zones: lowlands, midlands and highlands. The total sample size was divided to the three strata proportionally to their population size. From each stratum, 6 kebeles were selected at random and the sample size for each stratum was distributed to the kebeles proportional to their population size. Ultimately 750 subjects were selected using systematic random sampling technique. The sampling frame for pregnant women was developed by having a house to house enumeration. Presumptive symptoms of pregnancy (ammenoria and/or change in the size of uterus) with subsequent pregnant urine test were used to diagnose pregnancy.

### Data collection method

A structured and pretested questionnaire used to assess potential determinants of ZD. The parts of the questionnaire on dietary diversity (DD) and household food insecurity level were adopted from Food and Nutrition Technical Assistance (FANTA) indicator guide for Household Dietary Diversity Score (HDDS) [20] and Household Food Insecurity Access Scale (HFIAS) [21], respectively. Other parts of the tool were developed by the principal investigators (PIs). The content validity of the questionnaire was assessed against the conceptual framework of the study. Reliability of the tool was checked using test-retest method. Questions with less than 0.7 kappa or Pearson coefficient values were removed or revised.

Three trained and experienced enumerators collected the data. Interviews were made at the nearby health posts. The questionnaire was administered in local language. Height and weight were measured using calibrated Seca^® ^scales and the measurements were registered to the nearest 0.1 cm and 0.1 kg, respectively. Altitude of the kebeles measured using Magilan^® ^GPS system.

### Blood sample collection, serum extraction and zinc level determination

Venous blood was collected using plain SARSTEDT Monovette^® ^system and stainless steel needles. The blood was allowed to clot for 20 min and consecutively centrifuged at 3000 × g for 10 min. Visibly hemolyzed samples were discarded. Serum was extracted and transferred immediately into screw-top vials. The samples were kept and transported in icebox. The same day the samples were stored frozen at-20°C. Serum zinc concentration was determined at Ethiopian Health and Nutrition Research Institute using Varian SpectrAA^® ^Flame Atomic Absorption Spectrometer. Zinc deficiency was defined as a serum zinc level of less than 56 μg/dl during the first trimester, or less than 50 μg/dl during the second or third trimester [22]

Hemoglobin level was determined at the field using HemoCue Hb 301^®^. Anemia was defined as a hemoglobin level of less than 11.0 g/dl during the first or third trimester or less than 10.5 g/dl during the second trimester [23].

C-Reactive Protein (CRP) determined qualitatively using HumaTex CRP^®^.

### Data analysis

Data entry, screening and analysis were carried out by the PIs using SPSS 19.0. Descriptive analysis was done using mean, frequency and percentage. Independent *t*-test and one-way Analysis of Variance (ANOVA) used to compare serum zinc levels across categories of independent variables. The assumptions of ANOVA (normal distribution and homoscedasticity of the dependent variable across the categories of the independent variables) were checked to be fulfilled.

Wealth index quintiles (poorest, poorer, middle, richer, and richest) were computed using Principal Component Analysis (PCA). The index was calculated based on ownership of selected household assets, size of agricultural land, quantity of livestock and materials used for housing construction. PCA was also applied to reduce variables pertaining to maternal workload.

Logistic and linear regression analyses were used to control potential confounders. Independent variables which significantly associated (*P *< 0.05) to the dependent variable in simple regression models were exported to a multiple regression model for adjustment. In addition conceptually important confounders (like CRP status) were also adjusted. The major assumptions of logistic regression analysis (absence of multicollinearity and interaction among independent variables) and linear regression analysis (normally distributed error terms, linear relation between dependent and independent variables, homoscedasticity and absence of multicollinearity) were checked to be satisfied. The fitness of logistic and linear regression models were assessed using Hosmer-Lemeshow statistic and adjusted R squared value, respectively.

Hemoglobin values were adjusted for altitude according to the formulae recommended by Center for Disease Prevention and Control (CDC) [24].

### Ethical considerations

The study was conducted in confirmation of national and international ethical guidelines for biomedical research involving human subjects. Ethical clearance was obtained from the institutional review board of Addis Ababa University. Informed written consent was taken from the study subjects. Needle safety procedures were in line with WHO standard. Nutrition education was given to all subjects. Anemic women were given iron-folate supplementation.

## Results

### Background information about study subjects

Of 750 pregnant women recruited, 700 were willing to take part in the study. Accordingly, the response rate was 93.3%. The altitude of the kebeles included in the study ranged from 1584 to 2763 m Above Sea Level (ASL). Nearly half, 357 (51.0%), of the study participants were sampled from the midlands (1750 to 2300 m ASL); whereas, the remaining 187 (26.7%) and 156 (22.3%) were from highlands (> 2300 m ASL) and lowlands (< 1750 m ASL), respectively.

The mean age (+/-standard deviation) of the respondents was 28.5 years (+/-5.5 years). About two-third, 462 (66.0%) were illiterates and more than four-fifth, 561 (80.1%), were housewives. The vast majority were *Sidama *in ethnicity and Protestant Christians in religion. The median monthly household income was 200 birr. The average household size was 4.3 (+/-1.8). The average size of agricultural land per household was 0.38 (+/-0.19) hectares. (Table 1)

**Table 1:** Socio-demographic characteristics of study subjects, rural Sidama, Southern Ethiopia, Feb 2011

Variables	Frequency (*n *= 700)	Percentage
Age (years)		

15-24	158	22.6

25-34	425	60.7

≥ 35	117	16.7

Educational status		

Illiterate	462	66.0

Informal education	4	0.6

1st-4th grade	123	17.6

Higher than 4th grade	111	15.9

Occupation/Livelihood		

Housewife	561	80.1

Petty-trade	84	12.0

Farmer	44	6.3

Others	11	1.6

Ethnicity		

Sidama	679	97.0

Others	21	3.0

Religion		

Protestant	596	85.1

Catholic	60	8.6

Muslim	30	4.3

Orthodox	14	2.0

Marital status		

Married/Living together	696	99.4

Others	4	0.6

### Prevalence of zinc deficiency

The mean serum zinc concentration was 52.4 (+/-9.9) μg/dl (95% CI: 51.6-53.1 μg/dl). The value ranged from 26.5 to 77.9 μg/dl. Serum zinc levels for the first, second and third trimesters were 57.2 (+/-9.4), 53.6 (+/-9.8) and 50.1 (+/-9.9) μg/dl, respectively. ANOVA detected significant global difference across the three trimesters *(F = 20.170, P = 0.000) *and between all possible combinations of pared values. Based on simple linear regression analysis, a unit increment in trimester was associated with 3.46 μg/dl decline in serum zinc level *(t = 5.682, 0.000)*.

About 53.0% (95% CI: 49.3-56.7%) of the pregnant women were zinc deficient. The prevalence of ZD during the first, second and third trimesters were 46.8, 48.5 and 58.0%, respectively. Even after applying trimester specific cutoff points, women during the third trimester had 1.57 (95% CI: 1.29-1.91) times higher risk of ZD compared to those in the first trimester.

### CRP, fasting status and time of sample collection

According to IZiNCG, serum zinc concentration is artificially affected by the presence of acute inflammation/infection, time of day of blood sample collection and the fasting status of the subjects [2,22]. In this study the association of the aforementioned three factors with serum zinc level was assessed. Fasting was defined as no intake of food or beverage in the preceding 8 h of the sample collection [22]

About 8.4% of the samples were positive for CRP test. The mean zinc levels for CRP positive and negative samples were 49.8 (+/-9.6) and 52.6 (+/-9.9) μg/dl, respectively. The difference was statistically significant *(t = 2.159, P = 0.034)*. The mean zinc levels for samples collected in the morning and afternoon were 53.2 (+/-9.9) and 50.1 (+/-10.0) μg/dl, respectively. The difference was also significant *(t = 3.755, P = 0.000)*. Nevertheless, the mean zinc concentrations for fasting and non-fasting subjects were 52.3 (+/-9.9) and 52.9 (+/-9.9) μg/dl respectively and the difference was not significant *(t = 1.761, P = 0.079)*.

### Socio-demographic factors and zinc deficiency

Maternal age was negatively associated to zinc status. The mean serum zinc levels for women aged 15-24, 25-34 and 35-49 years were 53.6 (+/-10.2), 52.2 (+/-9.9) and 51.4 (+/-9.5) μg/dl, respectively. The global difference across the groups was significant (*F = 3.819, P = 0.022*). Compared to pregnant women aged 15-24 years, those aged 25-34 and 35-49 years had 1.57 (95% CI: 1.04-2.34) and 2.18 (95% CI: 1.25-3.63) times higher risk of ZD.

Maternal education and zinc status were positively associated. The mean serum zinc values for illiterates, those who completed 1st-4th grade and beyond 4th grade were 51.6 (+/-9.9), 52.6 (+/-9.5) and 54.9 (+/-9.5) μg/dl, respectively. The global difference was significant (*F = 4.728, P = 0.009*). Compared to women educated beyond 4^th ^grade, illiterates and those who completed 1st-4th grade were at higher risk of ZD with AOR of 1.71 (95% CI: 1.09-2.60) and 1.69 (95% CI: 1.01-2.85).

Household wealth index was not associated to zinc status. Nevertheless, women who were not involved in Income Generating Activities (IGA) had 1.74 (95% CI: 1.11-2.74) times higher risk of ZD than their counterparts.

The associations between ZD and geographical factors like altitude, agro-ecology and kebele were explored. There was no significant difference in serum zinc level across the 18 kebeles *(F = 0.442, P = 0.954)*. Similarly, altitude was not correlated to serum zinc level *(r = 0.041, P = 0.277)*. The mean serum zinc level and risk of ZD were not significantly different across the three agro-ecological zones (Table 2).

**Table 2:** Zinc deficiency and various socio-demographic variables among pregnant women in rural Sidama, Southern Ethiopia, Feb 2011

Variables	Serum zinc μg/dl (mean+/-sd)	ZD+	ZD-	Crude OR (95% CI)	Adjusted OR (95% CI) **
Age (years) (*n *= 700)					

15-24	53.6 (10.2)	66	92	1^r^	1^r^

25-34	52.2 (9.9)	232	193	1.68 (1.14-2.47)*	1.57 (1.04-2.34)*

≥ 35	51.4 (9.5)	73	44	2.31 (1.38-3.89)*	2.18 (1.25-3.63)*

Educational status (*n *= 700)					

Illiterate	51.6 (9.9)	255	207	1.74 (1.14-2.65)*	1.71 (1.09-2.60)*

1st-4th grade	52.6 (9.5)	70	57	1.74 (1.05-2.90)*	1.69 (1.01-2.85)*

Higher than 4th grade	54.9 (9.5)	46	65	1^r^	1^r^

Involvement in IGA (*n *= 700)					

No	52.1 (9.9)	333	272	1.84 (1.18-2.86)*	1.74 (1.11-2.74)*

Yes	53.8 (10.2)	38	57	1^r^	1^r^

Household wealth index (*n *= 700)					

Poorest	52.6 (9.6)	73	66	1.00 (0.62-1.60)	

Poorer	51.4 (10.3)	74	54	1.24 (0.77-2.01)	

Middle	53.1 (10.2)	78	74	0.93 (0.59-1.47)	

Richer	51.9 (9.4)	72	68	0.96 (0.60-1.53)	

Richest	52.7 (10.1)	74	67	1^r^	

Agro-ecology (*n *= 700)					

Highland	52.9 (10.3)	105	82	0.99 (0.64-1.51)	

Midland	52.6 (9.6)	179	178	1.25 (0.86-1.83)	

Lowland	52.9 (10.1)	87	69	1^r^	

1^r ^set as reference, * Significant association at 95% confidence level, ** adjusted for all significant variables and CRP status

### Zinc deficiency and parity

The mean parity was 2.4 (+/-1.7). The mean zinc level for nulliparas was 54.4 (+/-9.8) μg/dl. The corresponding value for parity categories of 1-2, 3-4 and 5 or more were 53.4 (+/-9.3), 51.8 (+/-10.1) and 47.1 (+/-9.8) μg/dl, respectively. Based on ANOVA, the global difference across the groups was significant *(F = 10.481, P = 0.000)*. Post-hoc test identified that those women with 0, 1-2 and 3-4 parity had significantly higher mean serum zinc values compared to grand multiparas. In the logistic model, grand multiparas were found to have 1.7 times higher risk (95% CI: 1.09-3.23) of ZD compared to nulliparas.

Among 553 who gave at least a birth in the previous 5 years of the survey, the association between birth interval and zinc status was assessed. In 55 (7.9%) of the cases the birth interval was less than the recommended 24 months. The mean serum zinc levels for women with short (< 24 months) and optimal (≥ 24 months) birth intervals were 49.3 (+/-7.8) and 52.2 (+/-10.1) μg/dl, respectively. The difference was significant *(t = 2.547, p = 0.013)*. In logistic regression model, the risk of ZD for women with short birth interval was 1.97 (95% CI: 1.07-4.24) times higher than their counterparts. (Table 3)

**Table 3:** Zinc deficiency and reproductive health factors among pregnant women in rural Sidama, Southern Ethiopia, Feb 2011

Variables	Serum zinc μg/dl (mean+/-sd)	ZD+	ZD-	Crude OR (95% CI)	Adjusted OR (95% CI)**
Parity (*n *= 700)					

0	54.4 (9.8)	52	64	1^r^	1^r^

1-2	53.4 (9.3)	138	138	1.23 (0.80-1.90)	0.72 (0.46-1.15)

3-4	51.8 (10.1)	126	105	1.48 (0.94-2.31)	0.77 (0.48-1.24)

≥ 5	47.1 (9.8)	55	22	3.08 (1.66-5.69)*	1.70 (1.09-3.23)*

Birth interval (*n *= 553)					

6-23 months	49.3 (7.8)	38	17	2.05 (1.13-3.72)*	1.97 (1.07-4.24)*

≥ 24 months	52.2 (10.1)	260	238	1^r^	1^r^

1^r ^set as reference, * Significant association at 95% confidence level, ** adjusted for all significant variables and CRP status

### Zinc deficiency and dietary factors

The majority, 451 (64.4%), of the respondents considered *Enset *(*Enset ventricosum*) as their staple diet; whereas, cereals (mainly maize) were considered by the remaining 249 (35.6%) of the respondents. The serum zinc levels in *enset *and maize staple diet categories were 53.3 (+/-9.0) and 50.8 (+/-10.5) μg/dl, respectively. The difference was significant *(t = 3.035, P = 0.003)*. After adjustments were made for other significant variables, the risk of ZD was 2.25 (95% CI: 1.61-3.16) times higher in maize based staple diet category.

The dietary diversity (DD) level was assessed using 24 h recall method. Respondents were asked whether they had taken any food from predefined 12 food categories in the previous day of the survey. Accordingly, the level of DD was computed out of the score of 12. The mean DDS was 4.3 (+/-1.7). More than one-third, 243 (34.7%), had low DDS. Compared to women with high DDS (DDS ≥ 6 [25]), the risk of ZD was 1.87 (95% CI: 1.02-2.91) and 2.57 (95% CI: 1.57-4.18) times higher among those with optimal (DDS = 4 or 5 [25]) and low DDS (DDS ≤ 3 [25]), respectively.

The mean serum zinc levels for women who consumed and who did not consume animal source in the previous day of the survey were 55.9 (+/-9.9) and 51.2 (+/-9.1) μg/dl, respectively. The difference was significant *(t = 6.088, P = 0.000)*. The risk of ZD was 2.51 (95 CI: 1.70-3.72) higher among those who did not consume animal source foods in the reference period.

The level of household food insecurity was assessed using HFIAS. The scale appraises the occurrence of nine food insecurity related events in the household in the preceding four weeks of the survey. The reporting of an event earned a score of one while the opposite earned zero. Accordingly, the scale was computed out of nine. The higher the score the more serious the household food insecurity problem. Those who scored above the mean value of HFIAS had 5.07 (95% CL: 3.67-6.99) times higher risk of ZD compared to women with below average score.

Coffee drinking is a common cultural practice in Sidama. In order to assess the association between coffee intake and zinc status, respondents were asked to estimate the volume of coffee they usually take (per 70 ml coffee cup) in a typical day. In average every woman takes 3.3 (+/-1.4) cups of coffee per day. Women who reported more than average coffee intake had 1.41 (95% CI: 1.06-1.84) times higher risk of ZD compared to their counterparts. (Table 4).

**Table 4:** Zinc deficiency and dietary factors among pregnant women in rural Sidama, Southern Ethiopia, Feb 2011

Variables	Serum zinc μg/dl (mean+/-sd)	ZD+	ZD-	Crude OR (95% CI)	Adjusted OR (95% CI) **
Staple diet (*n *= 700)					

Enset based	53.3 (9.0)	217	234	1^r^	1^r^

Cereal based	50.8 (10.5)	154	95	1.74 (1.28-2.40)*	2.25 (1.61-3.16)*

Diet Diversity (*n *= 700)					

Low	50.3 (9.6)	162	81	4.78 (3.04-7.51)*	2.57 (1.57-4.18)*

Optimal	52.0 (9.9)	168	150	2.68 (1.75-4.10)*	1.87 (1.02-2.91)*

High	56.9 (9.0)	41	98	1^r^	1^r^

Consumed animal source foods (*n *= 700)					

No	51.2 (9.1)	312	207	3.12 (2.18-4.45)*	2.51 (1.70-3.72)*

Yes	55.9 (9.9)	59	122	1^r^	1^r^

HFIAS (*n *= 700)					

Below the mean value	56.4 (8.7)	116	241	1^r^	1^r^

Above the mean value	51.2 (9.4)	255	88	6.02 (4.33-7.36)*	5.07 (3.67-6.99)*

Frequency of coffee intake per day (*n *= 700)					

≤ 3 coffee cups	53.1 (9.8)	189	141	1^r^	1^r^

> 3 coffee cups	51.6 (10.0)	182	188	1.38 (1.02-1.89)*	1.41 (1.06-1.84)*

1^r ^set as reference, * Significant association at 95% confidence level, ** adjusted for all significant variables and CRP status

### Zinc deficiency and maternal workload

In order to appraise the relationship between maternal workload and zinc status, the most common and laborious activities in the community were identified and the mean number of the activities in the preceding week of the survey were quantified. Using PCA, an index of five relative ordinal categories (lowest, lower, middle, higher, and highest) was generated. Based on ANOVA, the mean serum zinc level across the categories was not significantly different *(F = 0.509, P = 0.729)*. Similarly, in the logistic regression model the risk of ZD was not different across the maternal workload categories.

### Zinc deficiency, iron supplementation and anemia

Of all respondents, 140 (20.0%) took iron-folate supplement (daily dose of 150 mg ferrous sulphate and 500 μg folate) at least once in the preceding four weeks of the survey. However, only 105 (15.0%) reported full compliance for the supplement in the reference period.

In order to assess the association between iron supplementation and zinc status, the serum zinc level among those who did not take any supplement in the preceding four weeks was compared to those who too all the recommended 28 tablets. The mean zinc levels for the two groups were 52.9 (+/-9.8) and 50.9 (+/-10.0) μg/dl, respectively. The difference was not significant *(t = 1.966, P = 0.051)*. Similarly, using logistic regression the risk of ZD was not significantly higher in the iron-folate supplemented group (OR = 0.72 (95% CI: 0.47-1.11)).

The association between the duration of prenatal iron supplementation and zinc status was also assessed. The serum zinc levels for those who took the supplement for less than 4 weeks, 4-8 weeks and more than 8 weeks were 50.4 (+/-9.7), 52.9 (+/-10.2) and 49.6 (+/-10.2) μg/dl, respectively. The difference was not statistically significant *(F = 0.907, P = 0.406)*.

About two-third, 221 (31.6%), of the pregnant women were anemic. Anemia and ZD tend to occur together. About 149 (21.3%) of the subjects had both deficiencies. Among anemic women, more than two third, 149 (67.4%), were zinc deficient. Similarly, among zinc deficient women about 149 (40.3%) were anemic. After controlling other significant variables and CRP status, anemia and ZD were associated with OR of 2.37 (95% CI: 1.96-3.33).

### Zinc status and health care services

The serum zinc level and the risk of ZD were compared across different variables related to health care service. In logistic model, distance from nearby health facility, number of ANC follow up and receiving nutrition education during the pregnancy were not associated to ZD (Table 5).

**Table 5:** The association between zinc deficiency and health care related factors among pregnant women in rural Sidama, Feb 2011

Variable	Serum zinc μg/dl (mean+/-sd)	ZD+	ZD-	Crude OR (95% CI)
One-way walking distance from nearby health facility (*n *= 700)				

0-30 min	52.5 (9.9)	311	287	0.76 (0.50-1.16)

Longer than 30 min	51.6 (10.2)	60	42	1^r^

Frequency of ANC follow up (*n *= 700)				

0	53.4 (9.9)	184	180	1^r^

1-2	51.3 (9.8)	171	136	1.23 (0.48-2.20)

≥ 3	50.6 (9.5)	16	13	1.20 (0.56-2.58)

Received nutrition education during the pregnancy (*n *= 700)				

Yes	51.4 (10.2)	99	76	1.21 (0.86-1.71)

No	52.7 (9.8)	272	253	1^r^

### Linear modeling of maternal serum zinc level

Linear regression model containing all significant independent variables was developed in order to assess the total variability of the maternal zinc status explained by the covariates. The independent variables with their respective regression coefficients are given in Table 6.

**Table 6:** The outputs of the linear regression model

Variables	Unstandardized Coefficients	Standardized Coefficients	t	P value
Constant	42.816	-	12.417	0.000

Maternal age (in years)	-0.155	0.087	-2.410	0.016

Trimester (first, second, third)	-1.725	-0.106	-4.232	0.000

Parity	-0.753	-0.132	-3.581	0.000

Staple diet (0 = Enset, 1 = Maize)	-3.601	0.517	-6.971	0.000

Consumption of animal source food (0 = No, 1 = Yes)	0.983	0.475	2.069	0.039

DDS (0-12)	2.539	0.152	16.757	0.000

Frequency of coffee intake/day	-0.528	0.166	-3.192	0.001

Blood hemoglobin level	0.772	0.197	3.917	0.000

HFIAS (0-9)	-0.928	0.123	-7.523	0.000

Time of data collection (0 = Morning, 1 = Afternoon	-1.282	0.537	-2.386	0.017

The covariates explained 53.3% of the total variability of serum zinc level. Out of the explained variability the major share was attributable to nutritional factors (HFIAS, DDS, consumption of animal source foods, type of staple diet and frequency of coffee intake). The removal of the nutritional factors from the model would reduce the adjusted R squared value to 15.6%.

Unit increment in maternal age, gestational trimester, and parity were associated with 0.2, 1.7 and 0.8 μg/dl decline in serum zinc level, respectively. Net adjusted difference of 3.6 μg/dl was witnessed between *enset *and maize staple diet categories. The serum zinc level among those who consumed animal source food in the previous day of the survey was significantly higher by 1.0 μg/dl than their counterparts. DDS and HFIAS were associated to serum zinc level with unstandardized beta coefficients of 2.5 and-0.9, respectively. As the daily frequency of coffee intake increases by one cup, serum zinc declines by 0.5 μg/dl. Net adjusted difference of 1.3 μg/dl was witnessed between samples collected in the morning and afternoon. Serum zinc and blood hemoglobin level were positively associated with regression coefficient of 0.8.

## Discussion

The reported prevalence of ZD is prone to seasonality bias as the study is cross-sectional. The study was carried out in January and February which are locally considered as transitional months to household food insufficiency. If it had been conducted in food insecurity prone months (April to July), a higher prevalence would have been expected.

According to IZiNCG, the risk of ZD is considered to be of public health concern when the prevalence of low serum zinc concentrations is greater than 20% [26]. Hence, the study signals the public health significance of ZD in the area [1].

Till date two pocket studies in Ethiopia attempted to determine the prevalence prenatal ZD. Both were conducted in Sidama zone [6,13]. According to Abebe et al., in 2004 among 99 pregnant women in third trimester, the prevalence was 72% and the mean plasma zinc was 45.6 μg/dl [6]. According to Hambidge et al., in 2006 among 17 pregnant women in third trimester, the mean plasma zinc was 44.1 μg/dl [13]. The studies reported severe deficiency situation compared to the current study. The variation can be explained by the time gap and seasonal difference in data collection. Further, the studies may have overestimated the problem as they only included pregnant women in the third trimester.

IZiNCG suggested similar cutoffs point (50 μg/dl) for defining ZD during the second and third trimesters [2]. Nonetheless in the current study, parallel to studies conducted elsewhere [10,27], the zinc level in the third trimester was significantly lower than that of the second trimester. This is also consistent with the understanding that hemodilution continues into the third trimester [7,28]. The finding may indicate the need of having different cutoff point for the second and third trimesters.

According to IZiNCG, serum zinc level is artificially affected by CRP status, the time of day of blood sample collection and the fasting status the study subjects [2,22]. However, in current study fasting status was not associated to serum zinc level. Similarly, the serum zinc level differences between CRP positive and negative samples and samples collected in the morning and afternoon were not as huge as observed in NHANES II [22]. The finding signifies that in predominantly zinc deficient community, the effect of the aforementioned three factors may not be as prominent as expected in affluent community.

In the linear regression model the unexplained 46.5% of the variability in serum zinc level might be attributable to range of factors like serum copper level, serum albumin, intestinal and hemoparasites etc. which were not measured in the study.

Pregnant women from *enset *staple diet category were better-off in their zinc status as compared to women from maize staple diet category. The association could not be explained by dietary diversity and agro-ecological factors as these potential confounders were statistically controlled. The difference can be due to the better bioavailability of zinc in *enset *based diets [29].

Superior household economic standing enhances maternal zinc status [8,11]. However, in this study household wealth index was not associated to zinc status. Nevertheless involvement of women in IGAs is found to have positive influence. This might be due to the reason that in Ethiopia maternal income is usually directly spent to cover household food expenditures.

Two previous studies in Nigeria [8,30] failed to witness any definite trend on the effect of maternal education on prenatal zinc status. However, in the current study maternal education showed positive influence. Higher education status might have contributed to superior zinc status through enhancing good nutritional awareness and practice prior to and during pregnancy.

Maternal age was inversely associated to zinc status. The finding is consistent with the understanding that serum zinc level reaches peak during adolescence and young adulthood, and then declines [31]. Other studies also reported more or less parallel finding [8,10].

The study witnessed the deleterious effect of too many and too close births on zinc status. The finding is parallel to the knowledge that repeated pregnancies deplete maternal store. Previous studies conducted in Malawi [7], Nigeria [8] and USA [32] also supported the finding.

Laboratory and animal model studies indicated that zinc and iron compete for absorption in the intestinal lumen as they have similar physicochemical properties [33-35]. However, many community based studies concluded divergently [10,36-40]. In our study, parallel to studies conducted in Nigeria [37], Iran [38] and UK [39,40], daily iron-folate supplementation was not associated to maternal zinc status. This might be due to the reason that in the study community the intake of iron and zinc rich foods was low. Hence, competitive absorption which happens at higher concentration of the nutrients might not have taken place.

Hemoglobin and serum zinc levels were positively correlated. The association persisted after adjustments were made for potential nutritional and non-nutritional confounders. Many previous studies concluded likewise [29,41-43]. As the study is cross-sectional, it is not viable to exclude "the chicken or the egg" causality dilemma. However, as zinc is known to participate in multiple metabolic pathways, it might have causal role in anemia.

Chronic overexertion is a predisposing factor to maternal nutritional depletion [44]. However in current study maternal workload was not associated to zinc status. The finding might not be conclusive as level of maternal workload was measured using a relative rather than an absolute scale.

The study found negative association between frequency of coffee intake and zinc status. Coffee is known to contain tannin which can potentially inhibit zinc absorption [45]. However, empirical evidences are lacking. Few available animal model studies concluded divergently [46,47]. Further studies should be conducted in this direction.

Health care service related factors like distance from nearby health facility, frequency of ANC and nutrition education during pregnancy were not related to zinc status. This might be due to the reason that nutritional care is not well integrated into maternity services. In addition, the provision of nutrition education might not be effective in the absence of concurrent livelihood promotion strategies.

## Conclusions

Zinc deficiency is of public health concern in the study area. Most of the variability in serum zinc was explained by nutritional factors. Food insecurity, low dietary diversity, dependency on maize staple diet and plant source foods are key predisposing factors to zinc deficiency. Maternal education and involvement in IGAs have affirmative effect. Grand multiparity, old age pregnancy and frequent consumption of coffee were negatively associated to zinc status. Positive association was witnessed between serum zinc and hemoglobin levels. Prenantal iron-folate supplementation does not have negative effect on maternal zinc status. Access to health facilities, ANC follow up and nutrition education were not associated to zinc deficiency.

## Recommendation

National level zinc prevalence study should be conducted in Ethiopia. The high prevalence of ZD in the area must be combated through a range of short, medium and long-term strategies. Use of household based phytate reduction techniques can be considered as a short term option; whereas, implementation of agricultural based strategies like dissemination and use of zinc efficient maize strain and zinc containing fertilizers can be taken as medium term strategies. Rural livelihood promotion and socio-economic empowerment of women will yield the long-term solution. Birth control will also have affirmative input. Nutrition intervention must be strongly inculcated into maternity services. The effect of coffee intake on zinc status should be further investigated. The precise role and contribution of zinc to anemia should be studied.

## Competing interests

The authors declare that they have no competing interests.

## Authors' contributions

SG, FE and MU participated in study protocol development, data collection, analysis and write-up. All authors read and approved the final manuscript.

## Pre-publication history

The pre-publication history for this paper can be accessed here:

http://www.biomedcentral.com/1471-2458/11/898/prepub
